# Loss to follow-up in “test and treat era” and its predictors among HIV-positive adults receiving ART in Northwest Ethiopia: Institution-based cohort study

**DOI:** 10.3389/fpubh.2022.876430

**Published:** 2022-09-29

**Authors:** Berihun Bantie, Awole Seid, Gashaw Kerebeh, Animut Alebel, Getenet Dessie

**Affiliations:** ^1^Department of Adult Health Nursing, College of Medicine and Health Science, Debre Tabor University, Debre Tabor, Ethiopia; ^2^Department of Adult Health Nursing, School of Health Science, College of Medicine and Health Science, Bahir Dar University, Bahir Dar, Ethiopia; ^3^Department of Pediatrics and Child Health Nursing, College of Medicine and Health Science, Debre Tabor University, Debre Tabor, Ethiopia; ^4^School of Public Health, Faculty of Health, University of Technology Sydney, Ultimo, NSW, Australia

**Keywords:** loss to follow up, HIV positive adults, test and treat strategy, predictors, Northwest Ethiopia

## Abstract

**Background:**

People living with HIV/AIDS are enrolled in lifelong Anti-Retroviral Treatment (ART) irrespective of their clinical staging as well as CD4 cell count. Although this “Universal Test and Treat” strategy of ART was found to have numerous benefits, loss from follow-up and poor retention remained a long-term challenge for the achievement of ART program targets. Hence, this study is aimed at addressing the much-needed effect of the test and treat strategy on the incidence of loss to follow-up (LTFU) in Ethiopia.

**Method and materials:**

An institution-based follow-up study was conducted on 513 adults (age ≥15) who enrolled in ART at a public health institution in Bahir Dar City, Northwest Ethiopia. Data were extracted from the charts of selected patients and exported to Stata 14.2 software for analysis. Basic socio-demographic, epidemiological, and clinical characteristics were described. The Kaplan–Meier curve was used to estimate the loss to follow-up free (survival) probability of HIV-positive adults at 6, 12, 24, and 48 months of ART therapy. We fitted a multivariable Cox model to determine the statistically significant predictors of LTFU.

**Result:**

The incidence density of LTFU was 9.7 per 100 person-years of observation (95% CI: 7.9–11.9 per 100 PYO). Overall, LTFU is higher in the rapid ART initiation (24% in rapid initiated vs. 11.3% in lately initiated, AHR 2.08, *P* = 0.004), in males (23% males vs. 14.7% females, AHR1.96, *P* = 0.004), in singles (34% single vs. 11% married, with AHR1.83, *P* = 0.044), in non-disclosed HIV-status (33% non-disclosed 11% disclosed, AHR 2.00 *p* = 0.001). Patients with poor/fair ART adherence were also identified as another risk group of LTFU (37% in poor vs. 10.5% in good adherence group, AHR 4.35, *P* = 0.001).

**Conclusion:**

The incidence of LTFU in this universal test and treat era was high, and the highest figure was observed in the first 6 months. Immediate initiation of ART in a universal test and treat strategy shall be implemented cautiously to improve patient retention and due attention shall be given to those high-risk patients.

## Introduction

HIV/AIDS is a major global public health concern, having claimed the lives of 36.3 million people so far (1). Despite the fact that there is no cure for HIV/AIDS, antiretroviral medication (ART) has been the major treatment and preventative option since the late 1980's (2). According to the UNAIDS 2021 report, ART has been attributed to a 31 and 47% global reduction in the incidence of new HIV infections and HIV/AIDS-related deaths (3), respectively. With regard to this, in the year 2014, UNAIDS launched the three “95-95-95” targets for 2030 for HIV/AIDS, which are directly linked to improving ART services (4). After 2 years of the UNAIDS ambitious goals, the World Health Organization (WHO) also developed the “Universal Test And Treat (UTT)” strategy that recommends rapid initiation of ART for all HIV-positive individuals regardless of clinical status or CD4 cell count (5). Rapid ART initiation has been found to be important for increasing ART access, ensuring maximal and long-term viral load suppression, restoring and preserving immune function, improving quality of life, preventing transmission, and reducing HIV-related morbidity and mortality (6–8). In this regard, access to ART has been rapidly expanded and about 73% of HIV-positive individuals have gotten access to ART globally (1, 3). Ensuring long-term follow-up and retention on ART, however, remains a major challenge for the success of ART program targets as well as UNAIDS' ambitious goals.

Loss to follow-up (LTFU) which is defined as the absence of an ART refill or follow-up for 3 months or longer from the last follow-up schedule continues to be a significant public health concern on ART programs (2, 9, 10). Low- and middle-income countries (LMICs) carried the world's highest burden of LTFU, whereby only 65–70% of patients were receiving ART after 36 months of initiation of ART (11). Additionally, the incidence of LTFU ranges from 7.1 per 100 persons per year of observation in India (12), 10.3 in South Africa (13), 26.3 in Malawi (14), and 7.5 (15) in Uganda, respectively.

Moreover, studies conducted in Ethiopia before UTT have shown that both the proportion and incidence of LTFU greatly vary across regions and health institutions. For instance, in Debre Markos, Gondar, Pawi, Hadiya, and Jigigia (Eastern Ethiopia), it reaches 10.5 per 100 PYO, 11.6 per 100 PYO, 11.9 per 100 PYO, and 26.6 per 100 PY, respectively (16–20). The above studies also showed that the incidence of LTFU increases exponentially over time. Indeed, while the currently implemented UTT strategy demonstrated numerous positive effects compared to the previous Pre–UTT era, its association with loss to follow-up is not adequately investigated globally, particularly in Ethiopia. However, few studies conducted at some LMICs such as South Africa (21, 22), Nigeria (23), Zimbabwe (24), and Uganda (15) revealed that rapid initiation of ART significantly increases the incidence of LTFU. Approximately 34 and 33% of patients in Nigeria and South Africa who initiated ART in this era, respectively, were lost from their routine follow-up within 12 months in Nigeria and 24 months in South Africa (21, 22). On the other hand, according to a study conducted in San Francisco, United States of America, no significant difference was observed in the incidence of LTFU between same-day initiation and late initiation of ART (25).

LTFU is the leading cause of morbidity, mortality, hospitalization, treatment failure, high burden of opportunistic infections (OIs), the emergence of drug resistance to HIV, and transmission of the virus to uninfected sexual partners among patients with HIV/AIDS (9, 10, 12, 15–17). The factors which contributed to the increased burden of LTFU were age, sex, educational status, marital status, occupation, HIV-disclosure status, functional status, time to ART initiation, WHO clinical stage, CD4 level of the patient, BMI status, types of ART regimen, level of drug adherence, Cotrimoxazole Preventive Therapy (CPT) utilization status, substance abuse level, and Isoniazid Preventive Therapy (IPT) utilization status (12, 14, 15, 20, 26–30).

To decrease the incidence of LTFU, interventions such as expanding ART service sites, monitoring patients through frequent phone calls, offering a client-centered appointment system, and lowering the pill burden have been suggested and are being implemented both regionally and worldwide (2, 10). Despite these efforts, Ethiopia has been recognized as one of the top 25 nations with the largest number of new HIV infections, which has been connected to an increase in the prevalence of LTFU (31). Hence, a sustained effort is required to provide updated information on the incidence and predictors of loss to follow-up when the test and treat strategy is implemented. Therefore, the aims of this study were to investigate the incidence of loss to follow-up in “test and treat era” and its predictors among adults receiving ART in Northwest Ethiopia.

## Methodology

### Study design and setting

**Figure 1:**
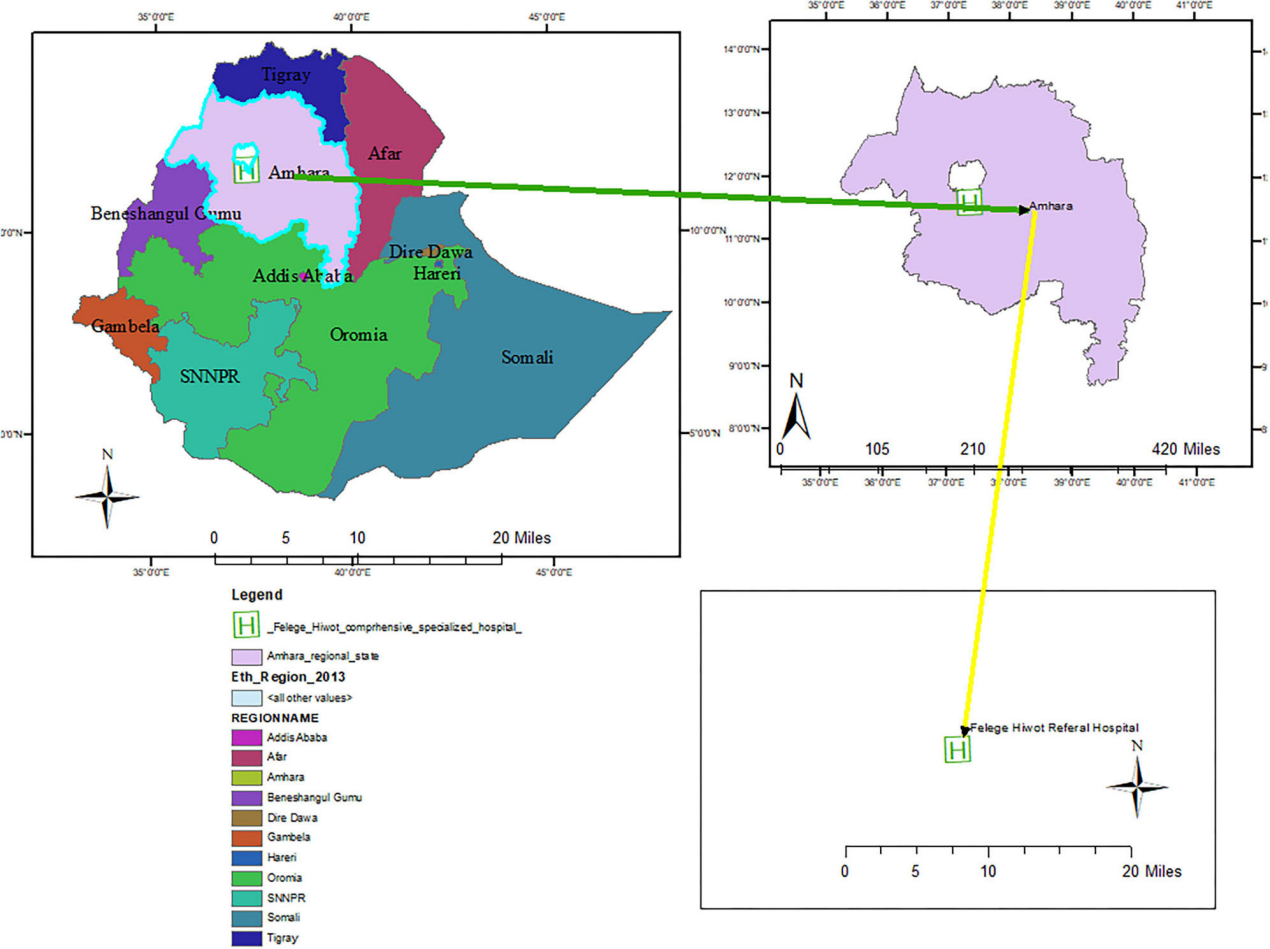
ArcMap of Felegehiwot Comprehensive Specialized Hospital, Northwest Ethiopia, 2022.

An institution-based cohort study was conducted on adults enrolled in ART in Felegehiwot Comprehensive Specialized Hospital (FHCSH), Northwest Ethiopia, from November 2016 to October 2020. It is one of the earliest comprehensive specialized hospitals in Amara regional state, located in Bahia Dar town, 565 km from Addis Ababa, the capital city of Ethiopia (Figure 1). The hospital is also the earliest ART service center in the region, where it has been providing ART care and service since 2003. According to the report of the Amhara regional health bureau, the hospital bears the highest burden of HIV/AIDS patient load in the region. A total of 13,275 HIV-positive adults have been enrolled in ART services since 2003, of which 1,030 newly started ART after the implementation of the universal treat-all strategy in November 2016. Currently, 6,645 people have active ART follow-ups in the hospital.

### Study period

This study took a 9-month duration starting from research question identification to the accomplishment of the overall work of the research. Data for the study were collected from 15 March 2020 to 15 April 2021 by three BSc nurse professionals. The advisors strongly participated in reviewing this work and providing constructive feedback from 29 October 2021 till 02 June 2021.

### Study participants

The source population for this study was all HIV-positive adults who were newly enrolled in ART at FHCSH following the implementation of the UTT strategy. All HIV-positive adults who were newly enrolled on ART from November 2016 to October 2020 at FHSCH and who had at least one active follow-up visit were included. On the contrary, HIV-positive adults whose charts were lost during the data collection period (six) and who had incomplete records of age, ART initiation date, and HIV confirmation date (25) were excluded from the study.

### Sample size determination and sampling technique

The minimum sample size required to conduct this study was calculated by STATA (V.14.2) software using a Log-rank test comparing two survival rate methods. By considering the following parameters, such as 0.05% level of significance, 80% power, 95% confidence interval, a 1:1 ratio of exposed to unexposed, and significant predictors of LTFU from a previous study (16), a 513 sample size was used to conduct the study. Initially, the medical registration numbers of HIV-positive adults were extracted from the electronic ART database. Then, a total of 513 study units were selected using a computer-generated random sampling method.

### Variables of the study

The outcome variable for this study is the occurrence of loss to follow-up at some point in time. The main exposure variable is the time to initiation of ART (within 7 or beyond 7 days). The other predictor variables were categorized as socio-demographic variables (sex, age, educational status, marital status, occupation, having cell-phone and residence) and Clinical and treatment-related factors including functional status, baseline body mass index (BMI) status, baseline CD4 count, presence or absence of OI, utilization of Isoniazid prophylaxis therapy (IPT), use of Cotrimoxazole preventive therapy (CPT), baseline WHO clinical stage, ART regimen type, history of ART regimen change and treatment failure, HIV disclosure status, and ART drug adherence status.

### Operational definitions

The outcome status of the patient was dichotomized into LTFU or censored. A patient is categorized as LTFU when an individual has failed to visit ART clinics more than 90 days after the last appointment date and has not been classified as either “died” or “transferring out” (10). Censored (non-LTFU) was considered when patients on ART did not develop the outcome (including transfer out patients, death, and those still on ART even at the end of the follow-up period). Time to event is the time interval (in months) between ART initiations and the occurrence of LTFU. Time to ART initiation is the time interval in a day from the confirmation of HIV status till the patient is enrolled in ART. Rapid ART initiation is defined as the initiation of ART within 7 days of confirmation of diagnosis irrespective of WHO clinical staging as well as CD4+ cell counts. The functional status of the patient is categorized as “working” if the daily activities of people living with HIV/AIDS were not altered due to illness; “ambulatory” if the patient was not fully working but was able to do minor tasks at home, and “bedridden” when the patient remained in bed most of the time (10). ART drug adherence was defined as the percentage of ART drug dosage taken from a monthly dose, and it was classified as good, fair, or poor. Hence, good adherence was reported if equal to or >95%, fair if 85% to 94%, and poor if < 85% of the monthly doses were taken (10).

### Data collection procedures and data quality

The data abstraction checklist was adapted from the Federal Ministry of Health (FMOH) ART follow-up form, the patient intake, and monitoring formats after reviewing different articles. Three BSc Nurse Professionals collected the data while being closely supervised by a master of public health professional with experience in monitoring and supervising ART programs. Both data collectors and supervisors received one-day training about the purpose of the study, how to extract relevant data, and how to guarantee the confidentiality of patient information to ensure the quality of the data. Supervisors also reviewed the consistency and completeness of the data every day.

### Data processing and analysis procedures

After the data were coded, it was entered into Epi_data version 4.6 and simultaneously exported to STATA 14.2 software to undergo further statistical analysis. Missing data were handled and managed by the multiple imputations technique after checking the missing at random (MAR) patterns of the variables with the missing record. The imputation was computed with Multivariate Chained Equation (MVCE) model (32). Finally, sensitivity analysis was computed to compare if there are significant changes in the effect sizes between the original data and imputed data. Summary measures such as median with Interquartile Range (IQR) and proportions were used to summarize continuous and categorical variables, respectively. Moreover, tables and graphs were also used to present data. The survival data were summarized by the Kaplan–Meier curve and the Log-rank test. The Kaplan–Meier curve was used to determine the LTFU-free survival time at each specific period. Whereas, the log-rank test was used to compare LTFU-free survival time between categories of each explanatory variable.

After fitting the model, its goodness of fit was checked by the Cox–Snell residual plot. Indeed, the proportional hazard (PH) assumption of the model was verified by both graphical tests (Log-log survival probability plot and Schoenfeld residual plot) and the Schoenfeld residual PH test. Both bivariable and multivariable Cox regression models were fitted to identify the predictors of loss to follow-up, and those variables with a *p* < 0.05 in the multivariable analysis were considered significant predictors of loss to follow-up.

### Ethical considerations

Since the study was conducted retrospectively using a patient's medical record, the need for informed consent was waived by the Institutional Review Board (IRB) of Bahir Dar University, College of Medicine and Health Science with a protocol number of 058/2021. Instead, a supportive letter was sent from the IRB to the study setting. Then, a permission letter was obtained from the directors, followed by the ART clinic focal person of Felegehiwot Comprehensive Specialized Hospital (FHCSH). Besides, information in the data extraction tool was kept anonymous. The confidentiality of information was kept throughout the entire study process, and the information was used only for the study purpose.

## Result

### Results of socio-demographic characteristics

From a total of 513 calculated samples of HIV-positive adults, 507 (98.8%) were included for final analysis. Six (06) participants were dropped from being studied due to the loss of the patient's medical chart and ambiguity of records inside the chart. Slightly more than half 268 (52.86%), 246 (51.3%) of the individuals were females and married, respectively. The median age of the study participants was 32 years, with an interquartile range (IQR) of 27–40 = 13 years. Regarding the residence of the study participants, more than two-thirds (362, or 71.4%) were from urban areas, whereas more than one-fourth (141, or 27.81%) did not attend formal education (Table 1).

**Table 1:** Socio-demographic characteristics of HIV-positive adults receiving ART by ART initiation time in FHCSH, Northwest Ethiopia, 2022.

**Variables** **(*N* = 507)**	**Variables category**	**ART initiation time**		**Frequency**	**Percent**
		**Within 07 days**	**After 07 days**		
Sex	Male	117	122	239	47.14%
	Female	170	98	268	52.86%
Age	Age 15–24	45	28	73	14.4%
	Age 25–34	130	88	218	43.0%
	Age 35–45	87	80	167	32.9%
	Age >45	25	24	49	9.7%
Marital status	Married	138	108	246	51.3%
	Single	73	51	124	23.1%
	Divorced	60	49	109	21.5%
	Widowed	16	12	28	4.1%
Residence	Urban	141	116	362	71.4%
	Rural	70	49	145	28.6%
Educational status	No formal education	81	60	141	27.8%
	Primary education	71	64	135	26.6%
	Secondary	82	53	135	26.6%
	Tertiary and above	53	43	96	18.9%
Occupation	Employed	82	65	147	29.0%
	Daily laborer	72	31	103	20.3%
	Farmer	11	22	33	6.5%
	Housewife	44	31	75	14.6%
	Merchant	39	39	78	15.3%
	Student	15	11	26	5.1%
	Others	24	21	45	8.9%
HIV-positive family member	Yes	116	89	205	40.4%
	No	171	131	302	59.6%
Reside on catchment area	Yes	160	123	283	55.8%
	No	127	97	224	44.2%
Having registered cell-phone	Yes	253	201	454	89.5%
	No	34	19	53	10.5%

Others: includes individuals who had no job and drivers.

### Clinical, immunological, and treatment-related characteristic

More than half, that is, 287 (56.6%) of study participants were initiated to ART within 7 days of confirmation of diagnosis, of which 112 (39%) were initiated within the same day of diagnosis. According to the WHO clinical staging, nearly two-thirds (66.3%) of them were in clinical stage I or II diseases at baseline. The median BMI and CD4 values of the study participants were 20.07 (IQR 18–22) and 278 (IQR 121–451), respectively. Altogether, nearly 37% (90) of HIV-positive adults had a baseline CD4 values < 200 cell/mm^3^. In terms of OI, around 108 (21.3%) of those individuals had TB/HIV co-infection. Regarding ART drug adherence, more than two-thirds (359, 70%) had good adherence levels (Table 2).

**Table 2:** Clinical and treatment-related characteristics of adults receiving ART by Art initiation time in FHCSH, Northwest Ethiopia, 2022.

**Variables** **(*N* = 507)**	**Category**	**ART initiation time**		**Frequency**	**Percent**
		**Within 07 days**	**After 07 days**		
Baseline WHO clinical stage	Stage 1	121	58	179	35.3%
	Stage 2	93	63	156	30.8%
	Stage 3	56	62	118	23.3%
	Stage 4	17	37	54	10.7%
Baseline CD4 category	>500	76	28	104	20.5%
	From 200–499	124	89	213	42.0%
	< 200	87	103	190	37.5%
Baseline BMI category	Underweight	73	65	138	27.2%
	Normal BMI	186	142	328	64.7%
	Overweight	28	13	41	8.1%
Baseline functional status	Working	238	166	404	79.7%
	Ambulatory	40	42	82	16.2%
	Bedridden	09	12	21	4.14%
History of TB /HIV co-infection	Yes	40	68	108	21.3%
	No	247	152	399	78.7%
History of OIs	Yes	76	102	178	35.1%
	No	211	118	329	64.9%
Baseline ART regimen	1e (TDF_3TC_EFV)	206	159	365	72.0%
	1j (TDF_3TC- DTG)	63	32	95	18.7%
	Others	18	29	47	9.3%
History of treatment failure	Yes	12	12	24	4.7%
	No	275	208	483	95.3%
History of regimen change	Yes	68	64	132	26%
	No	219	156	375	74%
History of side effect	Yes	19	22	41	8.1%
	No	268	198	466	91.9%
CPT prophylaxis status	Received	150	149	299	59.0%
	Not-received	137	71	208	41.0%
IPT prophylaxis status	Received	171	116	287	56.6%
	Not_ received	116	104	220	43.4%
Disclosure status	Yes	188	148	336	66.3%
	No	99	72	171	33.7 %
ART drug adherence	Poor /Fair	90	58	148	29.2 %
	Good	197	162	359	70.8%
**Cumulative percent of ART initiation time**	Rapid (within 07 days)		287	56.65
	After 07 days		220	43.4%

Others: Include 1c (AZT-3TC-NVP), 1d (AZT-3TC-EFV), 1f (TDF-3TC-NV) ART-drug regimen.

OIs, Opportunistic Infections; CPT, Cotrimoxazole Preventive Therapy; IPT, Isoniazid Preventive Therapy; TB, Tuberculosis.

### Outcome status of study participant

**Figure 2:**
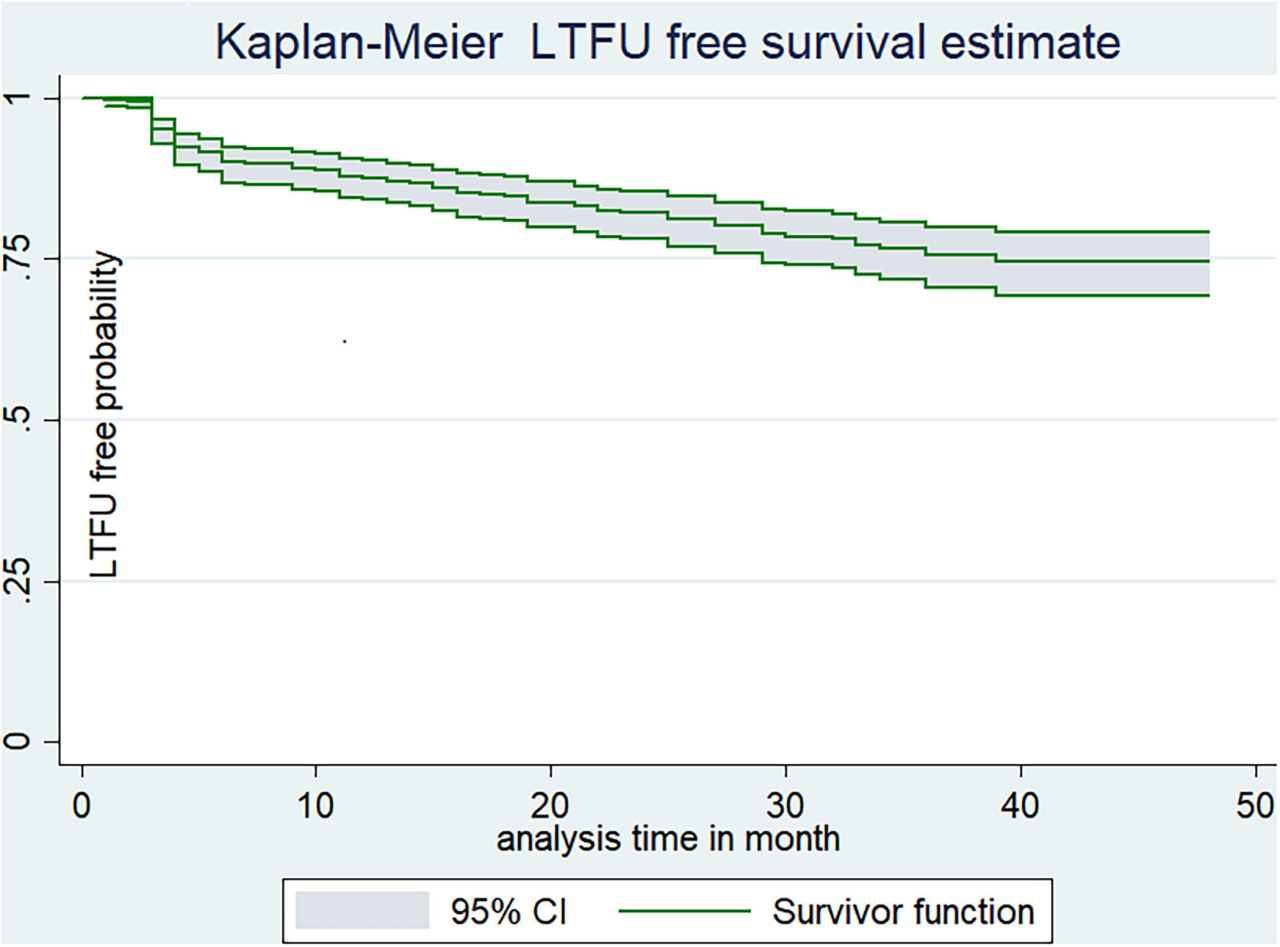
Kaplan-Meier estimate of LTFU-free estimate among adults receiving ART in FHCSH, Northwest Ethiopia, 2022.

The study participants were followed for a minimum of 1 month to a maximum of 48 months which provides a total of 11,493 person month (957.76 Person years of observation/PYO). The median follow-up time for the study participants was 23 months (IQR 9–36 months). Upon the compilation of the follow-up period, nearly one-fourth (18.34%, 95% CI: 15–22) of patients were LTFU, which yields the incidence density of LTFU as 9.7 per 100 (95% CI:7.9–11.9) PYO. Additionally, the incidence density of LTFU was 20.6 per 100 PYO (95% CI: 15.5–27.3), 13.6 per 100 PYO (95% CI: 10.5–17.7), 10.7 per 100 PYO (95% CI: 8.5–13.3), and 9.7 per 100 PYO (95% CI: 7.9–11.9), in the first 6, 12, and 48 months, respectively. Moreover, the estimated cumulative LTFU-free survival probability was 92% (95% CI: 88.8–93.7%) for the first 6 months, 87.9% (95% CI; 84.5–90.5%) at the end of 12 months, 82% (95% CI; 78–85.5%) at the end of 2 years, 76.6% (95% CI: 71.7–80.7%) at the end of 3 years, and 74.2% (95% CI: 68.6–88.8%) at the end of 4 years of follow-up period (Figure 2).

### Kaplan–Meier survival estimate based on variables

**Figure 3:**
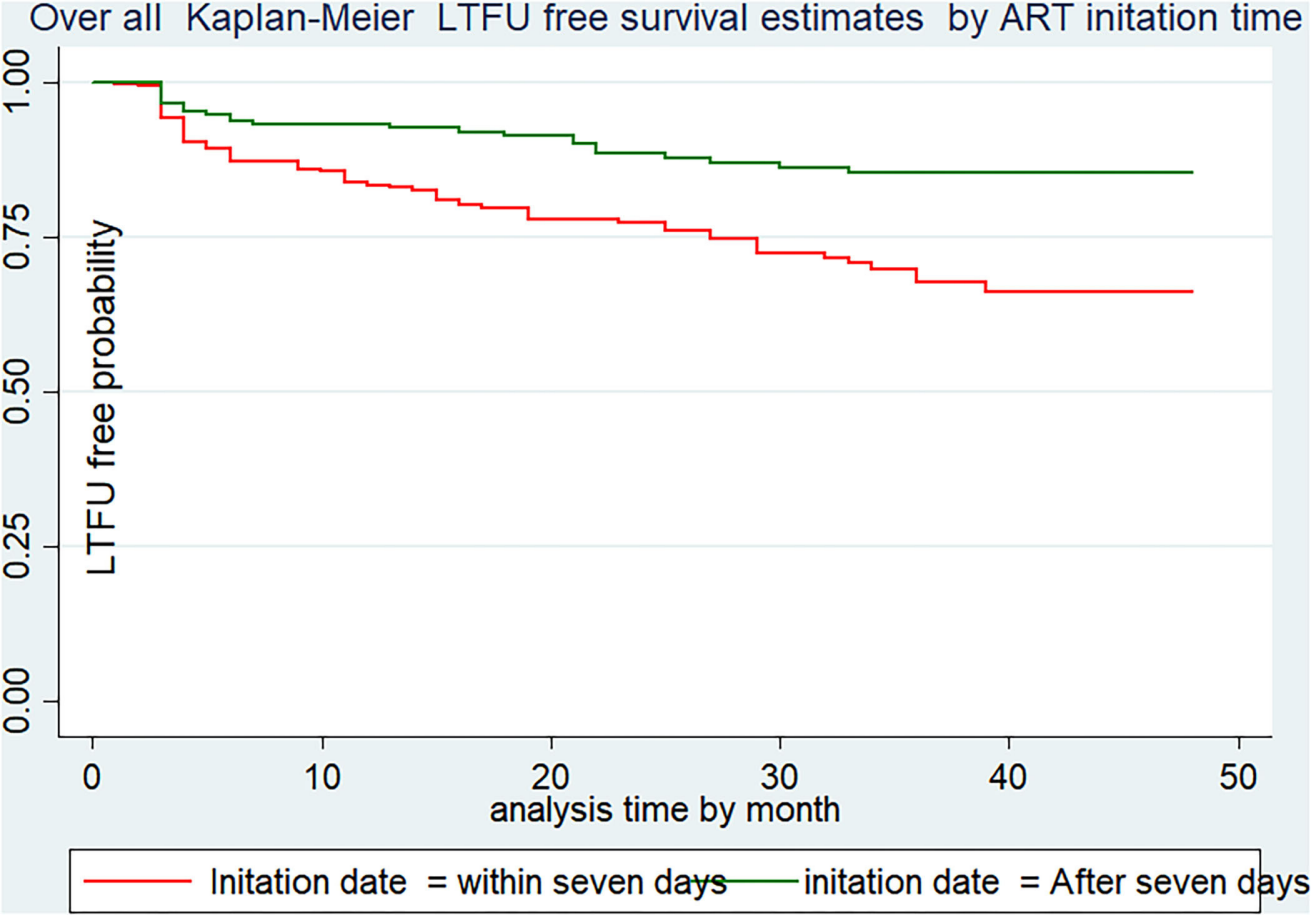
LTFU-free survival estimates using ART initiation date of Adults receiving in FHCSH, Northwest Ethiopia, 2022.

Both the incidence of LTFU- and LTFU-free survival probability vary across categories of each independent variable. For instance, the LTFU-free survival probability of patients who were initiated into ART within 7 days (65.8%, 95% CI: 57.61–72.9) was much lower than those who were initiated after 7 days of diagnosis (84.8%, 95% CI: 78.09–89.7). Additionally, the LTFU-free survival probability among patients who disclosed their status (84.19%, 95% CI: 78–88.5) was significantly higher than their counterparts (56.25%, 95% CI: 455–65.5) at the end of the follow-up period (Figure 3).

### Predictors of loss to follow-up

During bi-variable Cox-proportional regression analysis, 12 variables that had *p* < 0.25 were selected for multivariable Cox-regression regression analysis. In the final analysis, the following five variables namely time to ART initiation, sex, marital status ART disclosure status, taking CPT status, and ART drug adherence level were identified as the major of LTFU at *p* < 0.05. In this regard, HIV-positive adults who commenced ART within 7 days were almost two times more likely to be lost from their routine care than those who initiated later (24% in the rapidly initiated group vs. 11.3% in the lately initiated group with AHR:2.08, *p* = 0.004). Additionally, male HIV-positive adults have a nearly two-fold higher risk of LTFU than females (23% in male vs. 14.7% in female, AHR 1.96, value = 0.004). The hazard of LTFU among unmarried/single HIV-positive patients was also approximately two times that of married individuals (34% in single vs. 11% in married, with AHR 1.83, *P* = 0.044).

Moreover, the hazard of LTFU among HIV-positive adults who did not disclose their status is more than two times higher when compared to their counterparts (33% in non-disclosed vs. 11% in disclosed group AHR 2.00, *P* = 0.001). The risk of LTFU was 4.36 times greater in patients with fair/poor adherence than in those with good ART adherence (37% in poor adherence vs. 10.5% in the good adherence group, AHR 4.35, *P* = 0.000). In the end, HIV-positive adults who did not receive CPT were nearly two times at higher hazard of being lost from their follow-up compared to their counterparts (15% among received vs. 23.1%, AHR 1.74, *P* = 0.035) (Table 3).

**Table 3:** Bivariable and multivariable Cox regression analysis predictors of LTFU among adults on ART at FHCSH, Northwest Ethiopia, 2022.

**Variables** **(*N* = 507)**	**Variables** **category**	**Outcome status**		**CHR(95% CI)**	**AHR(95% CI)**
		**LTFU**	**Non-LTFU**		
Sex	Male	55	184	1.65 (1.09–2.50)*	1.96 (1.16–3.34)**
	Female	38	220	1	1
Age category	Age 15–24	28	45	3.05 (1.26–7.38)	2.5 (0.92–6.7)
	Age 25–34	36	182	1.11 (0.47–2.64)	1.60 (0.6–4.0)
	Age 35–45	23	144	0.94 (0.38–2.30)	1.46 (0.56–3.8)
	Age >45	6	43	1	1
Marital status	Married	26	220	1	1
	Single	42	82	3.79 (2.33–6.2)*	1.83 (1.01–3.29)**
	Divorced	17	92	1.47 (0.8–2.72)	1.13 (0.57–2.24)
	Widowed	8	20	2.96 (1.34–6.55)*	2.24(0.88–5.68)
Educational status	No formal Education	37	104	2.96 (1.43–6.13)*	1.36 (0.5–3.11)
	Primary education	21	114	1.67 (0.77–3.65)	0.91 (0.29–1.78)
	Secondary	26	109	2.15 (0.98–4.6)	1.23 (0.53–2.88)
	Tertiary and above	9	87	1	1
Occupation	Employed	19	128	1	1
	Daily laborer	28	75	2.38 (1.32–4.26)*	1.36 (0.68–2.71)
	Farmer	05	28	1.38 (0.5–3.68)	0.49 (0.3–1.52)
	Housewife	9	66	0.92 (0.41–2.04)	0.49 (0.3–2.04)
	Merchant	12	66	1.02 (0.49–2.01)	0.92 (0.43–2.16)
	Student	5	21	1.64 (0.61–4.40)	0.74 (0.24–2.28)
	Others	15	30	3.10 (1.57–6.10)*	1.34 (0.59–3.05)
HIV-positive family member	Yes	23	182	1	1
	No	70	232	2.18 (1.36–3.49)*	1.31 (0.76–2.25)
Time of ART initiation	Within 7day	68	219	2.34 (1.5–3.70) *	2.08 (1.26–3.4)**
	After 7 day	25	195	1	1
Disclosure	Yes	37	299	1	1
	No	56	115	3.26 (2.15–4.9)*	2.00 (1.4–3.5)**
Baseline CD4 category	>500	25	79	1	1
	From 200–499	41	172	0.72 (0.44–1.79)	0.78 (0.4–1.4)
	< 200	27	163	0.57 (0.33–0.98)*	0.77 (0.42–1.42)
CPT status	Received	45	254	1	1
	Not received	48	160	1.67 (1.11–2.5)*	1.74 (1.1–2.8)**
ART drug adherence	Fair/poor	55	91	4.79 (3.2–7.3)*	4.35 (2.7–7.01)**
	Good	38	323	1	1
History of drug side effect	Yes	14	30	1.71 (0.97–3.0)	0.58 (0.29–1.11)
	No	79	384	1	1

1-Reference category, ^*^statistically significant at bivariable with 5% level of significance, ^**^statistically significant at multivariable with 5% level of significance, CI- confidence interval.

**Figure 4:**
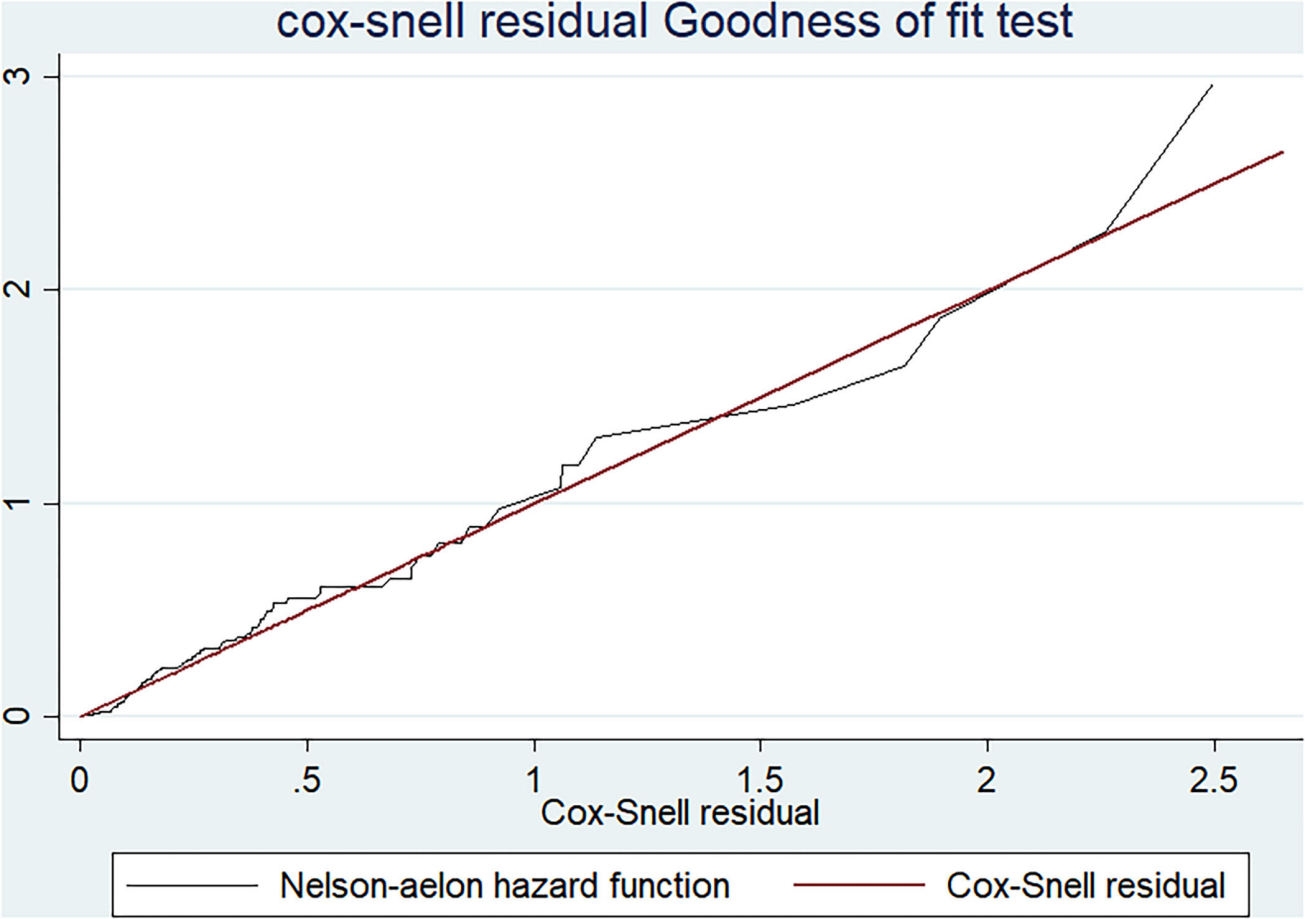
A graph of Cox-Snell residual test showing overall goodness of fit test result among adults receiving ART in FHCSH, Northwest Ethiopia, 2022.

The goodness of fit of the Cox proportional hazard regression model was checked by the Cox–Snell residual plot. Based on the Cox–Snell residuals plot, the Nelson_aelon hazard line has a 45-degree alignment with the reference line, indicating that the model is well-fitting (Figure 4). The proportional hazard assumption of the model was verified both graphically and through statistical tests (using the Log-Log survival probability plot, Schoenfeld residuals plot, and Schoenfeld residuals proportional hazard (PH) test), respectively. The proportional hazard assumption was satisfied since the *P*-value of the PH test for all variables in the final model was >0.05. Additionally, the yields of graphical tests (Log-Log survival probability plot and Schoenfeld residuals plot) illustrate that the effect of each predictor on the outcome was constant throughout time.

## Discussion

This institution-based retrospective cohort study was carried out to assess the incidence and predictors of LTFU following the implementation of the Test and Treat strategy at FHCSH, Northwest Ethiopia. In this study, the overall incidence density of LTFU among HIV-positive adults on ART at FHCSH was found to be 9.7 per 100 person-year of observation. The finding was in line with previous studies conducted in Gondar (16), Pawi (18), Hadiya zone (19), and Uganda (17). On the other hand, this finding is lower when compared with a study conducted at Karama hospital in the Somali region of Ethiopia (20). The possible discrepancy might be because of the mobile nature of the people in the Somali region as a result of their livelihood conditions (33). The presence of no stable settlement and the mobile nature of the people in turn lead patients on ART not to have regular follow-ups and eventually loss of care. In addition, access to ART will be burdensome for such mobile people, so they will end up with discontinuation of their care (34).

Socio-demographic characteristics such as sex and marital status were identified as important predictors of LTFU. For instance, it was observed that male HIV-positive adults had a nearly two-fold increased risk of LTFU compared to females. The findings were comparable to other studies conducted in eastern Ethiopia and India (12, 20, 30). The potential explanation for the observed link may be brought on by the fact that men are more likely to be drug users and travelers without stable dwellings, which ultimately causes them to lose from their routine follow-ups. Additionally, compared to married people, the risk of LTFU was nearly two times as high in single HIV-positive patients. This could be a result of single people not receiving enough social support from their families (35, 36).

In the current study, patients enrolled in ART rapidly were at an increased risk of being LTFU than their counterparts. This finding was supported by studies conducted in Uganda (15), South Africa (21), and Nigeria (23). This could be explained by the fact that since ART is a lifelong medication, it needs strong counseling, reassurance, and emotional readiness before starting it. Patients who begin ART rapidly, on the other hand, may not have enough time to receive a thorough assessment, adequate counseling, and information about the overall implications of early ART initiation and the need for a life-long ART treatment program (37, 38). Additionally, based on a study conducted in Japan, 50% of ART-associated immune reconstitution inflammatory syndrome (IRIS) occurred within the first month of ART initiation (39). The occurrence of SIRS on those who are not emotionally ready and who are relatively well initially will negatively influence patients' value of ART and ultimately cause them to lose their care. Moreover, the above finding was not in agreement with a study conducted in Taiwan (40) and San Francisco, United States of America (26). The disparity in results could be attributed to differences in socioeconomic status (education, occupation, and financial burdens) between the aforementioned countries and Ethiopia, which has a strong association with the incidence of LTFU (41).

HIV-positive patients who did not disclose their status to at least one individual were shown to be two or more times more at risk of being lost from their routine care than their counterparts. This finding is parallel with studies conducted in Jigiga (20) and a systematic review study conducted in LMICs (26). The reason behind this could be that individuals who do not disclose their status will not receive enough social support and build strong self-efficacy to have successful engagement in their routine care (29, 42).

This study also demonstrated that adherence to ART drugs was a significant predictor of LTFU. People with poor or fair ART adherence levels were more likely to become LTFU than people with high rates. This observation is consistent with research carried out in LMICs, and Malawi, Gondar, and Hadiya Zone Public Hospitals (14, 16, 19, 26). The strong link between adherence and loss to follow-up may account for the possible cause. Those factors which may contribute to poor adherence will indirectly result in ground losses of patients from their regular follow-up. Based on the findings of a qualitative study conducted in Uganda, the principal factors for poor adherence were poverty, presence of drug side effects, depression, poor peer support and counseling, and stigma and discrimination, which indirectly contribute to the increased incidence of LTFU, treatment failure, and death (38, 43).

Finally, patients who did not receive CPT through their follow-up period are more likely to experience LTFU than their peers. This finding was also comparable with a study conducted in Gondar and Nigeria (16, 23). The possible explanation for this association might be due to the effect of CPT on reducing the incidence of too many opportunistic infections such as pneumocystis pneumonia, toxoplasmosis, bacterial infections, and diarrheal diseases, which may indirectly improve the lived experience of the patient on ART (2). This implies that preventing the occurrence of opportunistic infections through widespread access to prophylaxis therapy enhances patients' retention in HIV care.

### Limitation of this study

This study has some limitations. Initially, due to the secondary nature of the data, it was initially quite challenging to examine the relationships between key crucial variables, including the mental status of the patient, provider and system-related issues, distance from the institution, and loss to follow-up. Second, due to poor tracing systems, patients who were leveled as LTFU might be died or self-transferred to other health facilities, which will overestimate the finding in this study. Additionally, since there are a lot of patients in this study who are referred to other health institutions with inadequate tracing, the finding of LTFU will be underestimated if those patients might be lost from care.

## Conclusion

The incidence of loss to follow-up was relatively high and it was higher among patients who start ART within 7 days. Moreover, a higher incidence of loss to follow-up was noted in the first 6 months of ART initiation. Additionally, not disclosing status, having poor/fair drug adherence, having ambulatory functional status, and not receiving CPT were identified as the predictors of loss to follow-up. Therefore, we strongly recommend the cautious implementation of the UTT strategy by complementing it with frequent follow-up, intensive counseling, and close monitoring for patients initiated rapidly. Health care providers would assure patient's willingness and emotional readiness before initiating ART.

## Data availability statement

All the data needed for this study are available in the manuscript, further inquiries can be directed to the corresponding author(s).

## Ethics statement

The studies involving human participants were reviewed and approved by Institutional Board of Bahir Dar University. However, since the study uses secondary data (patients' medical record) as a source of information, informed consent was waived by IRB of the university with the protocol number of 058/2021.

## Author contributions

BB: formulating research problem, design of study, data collection, analysis, interpretation, conclusion, and preparing initial manuscript draft. GD, AS, AA, and GK: actively participated in data collection, analysis and interpretation, and writing up the manuscript. All authors had thoroughly read and approved the manuscript.

## Conflict of interest

The authors declare that the research was conducted in the absence of any commercial or financial relationships that could be construed as a potential conflict of interest.

## Publisher's note

All claims expressed in this article are solely those of the authors and do not necessarily represent those of their affiliated organizations, or those of the publisher, the editors and the reviewers. Any product that may be evaluated in this article, or claim that may be made by its manufacturer, is not guaranteed or endorsed by the publisher.

## Abbreviations

AHR: Adjusted Hazard Ratio
AIDS: Acquired Immune Deficiency Syndrome
ART: Antiretroviral Therapy
BMI: Body Mass Index
CD4-Cluster Differentiation 4 cell
CPT: Cotrimoxazole Preventive Therapy
ESA: Eastern and Southern Africa
FHCSH: Felegehiwot Comprehensive Specialized Hospital
HIV: Human Immune Virus
IPT: Isoniazid Preventive Therapy
IR: Incidence Rate
LTFU: Loss To Follow-Up
LMIC: Low–and Middle-income countries
OIs: Opportunistic Infections
PYO: Persons Year of Observation
PLHIV: Peoples Living with Human Immune Virus
SSA: Sub-Saharan African country
TB: Tuberculosis
UNAIDS: Joint United Nations Program on HIV/AID
UTT: Universal Test and Treat strategy
WHO: World Health Organization.
